# On the relation between encoding and decoding of neuronal spikes

**DOI:** 10.1186/1471-2202-12-S1-P177

**Published:** 2011-07-18

**Authors:** Shinsuke Koyama

**Affiliations:** 1 Department of Mathematical Analysis and Statistical Inference, Institute of Statistical Mathematics, Tokyo, 190-8562, Japan

## 

Sensory information is represented in neuronal responses. Determining which code is used by the neurons is important for understanding how the brain processes the information [1].

Coding schemes used by neurons can be divided approximately into two categories. In rate coding, information about the stimulus depends solely on the firing rate, which is the average number of spikes per unit time. In temporal coding, on the other hand, there is significant correlation between the stimulus and any moments in the spike pattern having higher order than the mean [2].

While neural codes are characterized in terms of these encoding schemes, i.e., how the neurons encode the stimulus into the features of spike responses, experimentalists can access the neural codes only through decoding. From the decoding viewpoint, rate coding is operationally defined by counting the number of spikes over a period of time, without taking into account any correlation structure among spikes. Any scheme based on such an operation is equivalent to decoding under the Poisson assumption, because the number of spikes over a period of time, or the sample mean, is a sufficient statistic of the rate parameter of a Poisson process. Similarly, temporal coding can be defined by decoding the stimulus using a statistical model with a correlation structure between spikes (such as the m-IMI model, introduced below). If such a decoder improves on the performance of the rate decoder, it indicates that significant information about the stimulus is carried on the temporal aspect of spike trains [3].

We introduce a simple statistical model that has a correlation structure, taking the intensity function of a point process to be a product of two factors:(1)

where s_*_(*t*) represents the last spike time preceding *t*. The statistical model with the intensity function (1) has been called the multiplicative inhomogeneous Markov interval (m-IMI) model [4]. *ϕ*(*t*) is the free firing rate, which depends only on the stimulus, and *g*(*t*- s_*_(*t*)) is the recovery function, which describes the dependency of the last spike time preceding *t* and hence allows the m-IMI model to have a correlation structure between spikes. Note that (1) becomes the intensity function of a Poisson process if the recovery function is constant in time. It has been reported that the m-IMI model enhances decoding performance in real data analysis [3], which encourages use of the m-IMI model to test temporal codes.

Although neural codes can be defined in terms of either encoding or decoding, the resulting codes are generally different from one another. Here, we investigate the relation between the two viewpoints of neural coding in terms of rate and temporal coding schemes. Specifically, we investigate the extent to which decoders of each scheme decode rate and temporal codes that are defined in terms of encoding. Our main claim is that temporal decoding does not necessarily mean decoding a temporal code that the rate decoder fails to read, but also decoding certain rate codes with greater efficiency than the rate decoder.
